# Resource Cluster-Based Resource Search and Allocation Scheme for Vehicular Clouds in Vehicular Ad Hoc Networks

**DOI:** 10.3390/s24072175

**Published:** 2024-03-28

**Authors:** Hyunseok Choi, Yoonhyeong Lee, Gayeong Kim, Euisin Lee, Youngju Nam

**Affiliations:** 1 Research Institute for Computer and Information Communication, Chungbuk National University, Cheongju 28644, Republic of Korea; hschoi@chungbuk.ac.kr; 2 EW ELINT Technology R&D, LIG Nex1, Seongnam 13486, Republic of Korea; yoonhyeong.lee@lignex1.com; 3 School of Information Communication Engineering, Chungbuk National University, Cheongju 28644, Republic of Korea; kkyoung@chungbuk.ac.kr (G.K.); eslee@chungbuk.ac.kr (E.L.)

**Keywords:** VANETs, vehicular cloud, resource cluster, resource search, resource allocation

## Abstract

Vehicular clouds represent an appealing approach, leveraging vehicles’ resources to generate value-added services. Thus, efficiently searching for and allocating resources is a challenge for the successful construction of vehicular clouds. Many recent schemes have relied on hierarchical network architectures using clusters to address this challenge. These clusters are typically constructed based on vehicle proximity, such as being on the same road or within the same region. However, this approach struggles to rapidly search for and consistently allocate resources, especially considering the diverse resource types and varying mobility of vehicles. To address these limitations, we propose the Resource Cluster-based Resource Search and Allocation (RCSA) scheme. RCSA constructs resource clusters based on resource types rather than vehicle proximity. This allows for more efficient resource searching and allocation. Within these resource clusters, RCSA supports both intra-resource cluster search for the same resource type and inter-resource cluster search for different resource types. In RCSA, vehicles with longer connection times and larger resource capacities are allocated in vehicular clouds to minimize cloud breakdowns and communication traffic. To handle the reconstruction of resource clusters due to vehicle mobility, RCSA implements mechanisms for replacing Resource Cluster Heads (RCHs) and managing Resource Cluster Members (RCMs). Simulation results validate the effectiveness of RCSA, demonstrating its superiority over existing schemes in terms of resource utilization, allocation efficiency, and overall performance.

## 1. Introduction

In Intelligent Transport System (ITS), the autonomous vehicle technology is rapidly developing following the development of onboard units (OBUs) in the vehicles. Drivers will be relieved from driving vehicles when autonomous vehicles become commercially available in the near future. Accordingly, the need for applications focusing on entertainment as well as existing applications focusing on driving safety and convenience for drivers and passengers is emerging [1,2,3,4,5]. Vehicles generate and consume various contents of different types and sizes in VANETs. However, entertainment applications (e.g., OTT, YouTube, Netflix, etc.) are gradually becoming high-definition and large-capacity with the development of digital technology [6,7]. As a result, there is a limit to the production and consumption of entertainment content with the available resources of a single vehicle. To produce and consume valuable content such as high-definition and large-size contents, a vehicular cloud is needed so that multiple vehicles communicate with each other to make themselves a single entity with large resources by sharing their own resources [8,9,10,11].

Many schemes have been proposed for vehicular clouds. The vehicular cloud is constructed as flat and hierarchical architectures from an architectural perspective.

Vehicular clouds, which usually have a flat structure, are formed through V2X communication between entities that comprise the network on VANETs. They are broadly categorized into two types of clouds: V2I communication, which is vehicle-to-infrastructure communication, and V2V communication, which is vehicle-to-vehicle communication. The V2I cloud, in which the requester vehicle downloads the content from the Internet through a fixed infrastructure, uses V2I communication [10,12,13,14,15,16,17,18,19]. Since the requester vehicle only can connect to the Internet to download the content while the requester vehicle travels in the communication range of the fixed infrastructure, the requester vehicle cannot download the content when the requester vehicle gets out of the communication range of the fixed infrastructure. If the requester vehicle cannot download the whole content from the present fixed infrastructure, it requests the content at the next fixed infrastructure. To continue downloading the content, the requester vehicle sends the information of the content repeatedly to the next fixed infrastructure. This process is iterated until the requester vehicle downloads the whole content, and it causes a communication delay. Furthermore, the requester vehicle uses cellular communication to connect to the Internet, and, thus, the communication cost highly increases. To reduce communication delay and cost, V2V cloud construction was proposed. The V2V cloud is constructed by vehicles that have similar mobility and communicate with each other. The requester vehicle requests resource allocation to other vehicles to consume the content [20,21,22,23,24,25]. Since the V2V cloud travels as a set of vehicles without the connection of a fixed infrastructure, it can consume the content continuously and reduce communication costs. However, the existing V2I and V2V cloud schemes have several problems because they perform the resource search and allocation based on the flat structure. First, the number of neighbor vehicles that can provide available resources to the requester vehicle is limited by the mobility of vehicles such as the location, speed, and future travel route. Second, the V2V cloud construction requires a large number of computations because it is constructed based on the connection time among vehicles. Third, the single-hop V2V cloud has a limit because the communication range of vehicles is low [20]. To cover these limits, multi-hop V2V cloud construction schemes have been proposed [21,22,23,24,25]. However, as the hop count increases, the connectivity among vehicles becomes weak. In the worst case, the vehicular cloud cannot be constructed by the mobility of vehicles. These issues decrease vehicular cloud stability and service availability. This flat-structured vehicular cloud is formed and released on-demand according to the request of the requester vehicle, so the vehicular cloud can be destructed at any time as the vehicle’s mobility changes. This considerably reduces the stability of the cloud service used by the requester vehicle, and repeated vehicular cloud destruction increases communication delay and overhead. To solve these problems, some schemes have been proposed to support the construction of vehicular clouds utilizing a hierarchical structure using clustering techniques on VANETs. The clustering technique can be utilized in VANETs [22,26,27,28,29,30,31,32,33,34,35,36]. Some schemes have proposed the V2V cloud construction utilizing the clustering technique based on the hierarchical structure [22,26,30]. In [26,27,28], these schemes construct clusters using the vehicles traveling on the same travel route. These schemes search and allocate resources for the vehicular cloud. In [22], SERVitES divides the map into four sections and constructs each cluster. However, these schemes construct the cluster for managing the resource information of vehicles based on the connectivity among vehicles in the same load or area. Clusters within this architecture may lack sufficient resources to construct a vehicular cloud due to limitations in the numbers or resource availability of vehicles caused by the proximity constraint among vehicles. In this situation, the cluster should request resources from other clusters to find enough resources for the vehicular cloud. Since this inter-cluster communication for searching resources is based on multi-hop communications with many hop counts, the existing schemes cannot rapidly search and stably allocate resources for constructing vehicular clouds in VANET environments based on vehicles with various resource types and different states of mobility. As a result, the existing scheme cannot satisfy various vehicular cloud services. Therefore, it is necessary to propose a new cluster-based scheme to manage the resource information of vehicles to enable efficient resource search and allocation for supporting various vehicular cloud services.

Therefore, we propose a Resource Cluster-based Resource Search and Allocation (RCSA) scheme that exploits resource clusters based on resource types to efficiently search and allocate resources for supporting various services of vehicular clouds. Unlike the previous schemes, RCSA constructs vehicles with the same resource type as a cluster in order to easily search and effectively allocate resources for providing vehicular cloud services. To stably manage the resource information of cluster member vehicles in each resource cluster, a Cluster Header (CH) is selected as the member vehicle with the longest average connection time and the largest resource amount among them. Since a requester vehicle can belong to the resource cluster with the same resource type or with a different resource type, RCSA provides both an intra-resource cluster search for the same resource type and an inter-resource cluster search process for the different resource types, respectively. For the allocation of resources, RCSA uses vehicles with longer connection times and larger resource amounts in order to prevent the breakdown of vehicle clouds and to reduce the communication traffic within the vehicle clouds. Since any vehicle as a CH or a cluster member vehicle joins or leaves a resource cluster due to its mobility, RCSA provides a resource cluster reconstruction to replace the CH and to manage cluster member vehicles. Simulation results conducted in various environments verify that RCSA achieves better performance than the existing schemes in terms of the resource searching delay, the number of packets, and the success ratio. Our contribution is as follows:

- We propose the construction of resource clusters in a real field, where vehicles with different types of resources are traveling. These resource clusters are constructed based on both resource types to decrease the delay of resource search and the connection time between vehicles to increase the stability of resource clusters.
- The requester vehicle can quickly search for the desired type of resource in the resource clusters by utilizing intra- and inter-resource search and requesting an allocation of the available resource to construct the vehicular cloud.
- We evaluate the performance of our proposed scheme compared to the existing clustering scheme in terms of resource searching delay, number of packets, and success ratio. The main performance metric for the proposed scheme is the success ratio, which shows a 20 to 60 percent performance improvement compared to the existing scheme.

The remainder of this paper is organized as follows: first, we provide the related works on the proposed scheme for vehicular clouds in Section 2. Next, the network model and the overview of the proposed scheme are presented in Section 3. Then, we describe the proposed scheme in detail in Section 4. Next, simulation results are presented to evaluate the performance of the proposed scheme in Section 5. Finally, the paper is concluded in Section 6.

## 2. Related Works

In this section, we describe the related works for vehicular clouds in VANETs. Vehicular clouds can be categorized into flat and hierarchical structures. In a flat structure, a vehicle cloud is constructed on-demand through V2X communication with entities in VANETs (e.g., vehicles, Road-Side Units, servers, etc.). On the other hand, entities in VANETs form and maintain a network of a hierarchical structure regardless of the request of the requester vehicle. When a requester vehicle requires a cloud service, it can enter the communication range of this network and utilize the cloud service.

First, we survey vehicular clouds with a flat structure, which are categorized into two communication types: V2I cloud and V2V cloud. The V2I cloud, where the requester vehicle downloads content from the Internet through the RSU, utilizes V2I communication. Several schemes have been proposed for V2I cloud construction [12,13,14,16,17,19,37].

Mershad et al. [12] introduced a system leveraging Roadside Units (RSUs) as a cloud directory to store information on proximate vehicular cloud servers. The system employs a ’STAR’ (Service Target) for streamlined access to services and resources via RSUs. Through shared registration data, RSUs enable vehicles to explore and utilize services within designated areas. The system further aids in STAR selection based on user requirements, enhancing the Quality of Service in dense networks by optimizing communication speed between RSUs and the Cloud. Yu et al. [14] introduced a novel cloud architecture for vehicular networks, comprising a vehicular cloud, a roadside cloud, and a central cloud. This design seamlessly incorporates cloud computing into vehicular networks, facilitating the sharing of computation, storage, and bandwidth resources among vehicles. The three-layered architecture optimizes the organization of cloud resources, providing vehicles with resilient options for selecting cloud services. Despite central clouds offering abundant resources, they may encounter significant end-to-end communication delays. To address this, the authors applied a game–theoretical approach to the optimal allocation of cloud resources within this hierarchical framework. Salahuddin et al. [37] introduced a Cloud Resource Management (CRM) model designed to minimize reconfiguration overhead, service replications, and infrastructure delay while meeting network and link layer constraints. The proposed model outlines the RSU cloud architecture, incorporating micro data centers, and includes an analysis of reconfiguration overhead. The CRM design is formulated as multi-objective Integer Linear Programming (ILP). Additionally, the authors developed an efficient heuristic for CRM, optimizing parameters such as VM migrations, control plane overhead, number of service hosts, and infrastructure delay. Lin et al. [13] introduced a Semi-Markov Decision Process (SMDP) model for optimal resource allocation in vehicular cloud computing (VCC), integrating both Vehicle-to-Vehicle (V2V) and Vehicle-to-Infrastructure (V2I) interactions. This model aims to determine the most efficient strategy for VCC resource allocation. Two additional features were incorporated to enhance the SMDP model, resulting in diverse outcomes compared to the original version. The resource pool encompasses units from Roadside Units (RSUs) and accounts for the number of RSUs catering to multiple vehicle types. Additionally, the model accommodates various Poisson distributions to address the heterogeneity observed among different vehicle types. Lee et al. [19] introduced an RSU-aided scheme for vehicular resource search and cloud construction within Vehicular Ad Hoc Networks (VANETs). In this approach, RSUs collect data on the mobility and resources of vehicles, coupled with their location information within the RSU coverage area. Utilizing this information, RSUs identify provider vehicles capable of supplying the necessary resources for constructing a vehicular cloud requested by a specific vehicle. The criteria for selecting provider vehicles encompass the connection duration between each candidate vehicle and the requester vehicle, the resource size of each candidate vehicle, and the connection starting time to the requester vehicle. Jaiboon et al. [16] introduced a mechanism for infotainment data distribution in vehicular networks utilizing the RSU cloud. The proposed system includes models and algorithms for vehicles, the RSU cloud, and the data center. By harnessing the capabilities of cloud computing, the RSU cloud overcomes limitations associated with traditional RSUs, thereby improving the efficiency of distributing infotainment messages. This approach not only reduces communication delay but also enhances throughput for vehicle communication through the RSU cloud, streamlining the distribution of infotainment messages. Elahi et al. [17] introduced a dynamic resource allocation framework for a vehicular cloud scenario. Their approach features a vehicular cloud network with a fixed roadside unit acting as the coordinator. The authors devised three resource allocation algorithms, each tailored to specific priorities: partial prioritization for service cars, prioritization for local cars, and bandwidth reservation for partially serviced cars. The dynamic allocation of resources entails considering the maximum portion of resources among all competing vehicles. The study examines the trade-off between serving the maximum number of vehicles and ensuring the highest possible quality of service.

Next, the V2V cloud can be constructed among vehicles without any infrastructure. The requester vehicle can construct a vehicular cloud with the other vehicles after the requested resources are searched and allocated [20,21,22,23,25]. Many schemes have been proposed to construct the V2V cloud in VANETs. Meneguette et al. [21] introduced an innovative protocol for managing resources within the cloud, specifically targeting the resources present in vehicles. The protocol promotes cooperation and collaboration among vehicles, recognizing the substantial embedded resources within vehicles that can be shared without depending on external infrastructure for communication assistance. This protocol effectively enhances the availability of resources to vehicles, consequently boosting the capacity of resources that can be utilized within the vehicle cloud. Meneguette et al. [23] introduced a peer-to-peer protocol tailored to resource and service search and management within the vehicular mobile cloud, operating autonomously from external infrastructure. The scheme takes into consideration the dynamic nature of the vehicular cloud shaped by vehicles, characterized by mobility and frequent topology changes. The protocol places emphasis on vehicle mobility, recognizing its pivotal role in influencing resource search time, cloud construction, maintenance, and service duration. The protocol facilitates collaboration and cooperation among vehicles, empowering them to share resources across the vehicular network and identify hosts providing the desired services. Meneguette et al. [22] introduced a protocol aimed at simplifying the search and management of resources within a vehicular cloud, operating independently of support from roadside infrastructure. The proposed approach leverages idle resources within vehicles, enabling their sharing within a mobile cloud formed by a set of vehicles. To create this dynamic mobile cloud, clustering techniques are employed to form efficient groups of vehicles. The mobile cloud dynamically moves along the road, allowing vehicles to join or leave based on their proximity and speed. Choi et al. [25] introduced a multi-hop vehicular cloud construction scheme that utilizes a multi-hop connection, time-based intermediate vehicle selection strategy. This approach is tailored to a multi-hop resource search, with the objective of identifying vehicles possessing ample resources for constructing a multi-hop vehicular cloud. The primary aim is to minimize the likelihood of cloud failure during the construction process. Resource allocation for the vehicular cloud is determined based on the connection time between vehicles and the number of neighboring vehicles, leading to heightened service availability and a decrease in the number of signaling packets.

These vehicular clouds based on the flat structure have several problems. Since the mobility of the requester vehicle changes frequently and the RSUs are deployed in static positions, the connection time between the requester vehicle and RSU is very short. This reduces cloud service availability. Additionally, the requester vehicle needs to search for other vehicles that have limited resources within the large communication range to construct a V2V cloud. This causes weak connectivity and significant delays in cloud construction. To address the disadvantages of vehicular clouds based on the flat structure, vehicular cloud construction schemes using clustering have been proposed based on the hierarchical structure [22,26,27,28,29]. This vehicular cloud has the advantage that the requester vehicle does not need to search for resources directly because the cluster header manages the information of all vehicles participating in the cluster. Thus, the requester vehicle requests available resources when it meets the vehicle participating in the cluster. This reduces resource search and allocation delays, thereby reducing vehicular cloud construction delays. Zhang et al. [26] introduced a novel multi-hop clustering scheme to create stable vehicle groups. In this approach, a new mobility metric is introduced to represent the relative mobility between vehicles within the multi-hop distance. Vehicles determine the relative mobility with other vehicles by measuring packet transmission delay. Subsequently, cluster head nodes are selected based on the lowest aggregate mobility value, and other vehicles decide to join the cluster based on this criterion. This scheme is designed to establish stable vehicle clusters by considering the dynamic mobility interactions between vehicles within the multi-hop distance. Arkian et al. [27] introduced a novel vehicular cloud architecture that leverages a clustering technique for grouping vehicles and enabling collaborative resource sharing. The cluster structure’s flexibility is augmented through the integration of fuzzy logic in the cluster head selection process. Furthermore, resource management is enhanced by incorporating the Q-learning technique. This technique is employed to select a service provider from among the participating vehicles, thereby improving the efficiency of cluster head decisions. The proposed architecture seeks to optimize both resource utilization and decision-making processes within the vehicular cloud. Zhang et al. [28] introduced a novel passive multi-hop clustering algorithm (PMC) built on the principles of a multi-hop clustering algorithm to guarantee comprehensive cluster coverage and stability. The PMC algorithm prioritizes the selection of optimal neighboring nodes to join the same cluster during the cluster head selection phase, employing a priority-based neighbor-following strategy. Emphasizing cluster stability, the algorithm ensures the stability of cluster members and selects the most stable node as the cluster head within the N-hop range. This strategy substantially improves the overall stability of the clustering process. Wu et al. [29] introduced a clustering scheme incorporating the channel effect, considering both multipath fading in-vehicle communication and the Doppler effect due to the relative speed of vehicles. The initial focus is on cluster head selection for Vehicle-to-Infrastructure (V2I) communication, where the cluster head serves as a relay for other cluster members. This design is particularly suitable for the application of vehicles as a cloud platform, enabling connected vehicles and passengers to share information and computing resources. The cluster head, in this context, can function as the coordinator for the Vehicle-as-a-Service (VaS) platform, facilitating collaborative sharing and resource utilization within the vehicular cloud. Meneguette et al. [22] introduced an efficient clustering scheme aimed at establishing and maintaining an effective vehicular cloud. This approach assists in the search and management of resources for vehicular cloud construction without depending on support from roadside infrastructure. The cluster dynamically moves along the road, enabling vehicles to join or leave based on their proximity and speed. To support the mobility of clusters, a new gateway is selected to optimize the resource quality of service parameters. Additionally, a novel routing mechanism is designed to tackle the challenges associated with Vehicle-to-Vehicle (V2V) communication within this dynamic and mobile cluster environment.

In the related works, these schemes primarily evaluate performance in terms of delay, success ratio, and overhead. The performance of these metrics is sensitively affected during the vehicular cloud construction process due to the mobility of vehicles. However, these schemes do not consider the various resource types that a vehicle possesses, including storage, bandwidth, network, computing power, etc. Since the requested resource types vary for each requester vehicle, it is necessary to form clusters by dividing the resource types.

In this paper, we propose to construct a resource cluster considering the resource type of vehicles, allowing a requester vehicle to construct a vehicle cloud using the resource cluster. Our proposed scheme specifically focuses on constructing resource clusters among vehicles with the same type of resources. To evaluate the performance of our proposed scheme, we introduce a metric called resource searching delay. This metric measures the delay experienced by a requester vehicle when searching for the desired type of resource, in addition to the metrics typically evaluated by existing schemes.

## 3. Network Model and Scheme Overview

### 3.1. Network Model

**Figure 1:**
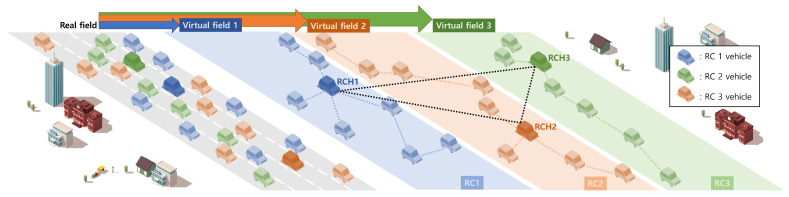
The network model of the proposed scheme involves vehicles with the same resource type virtually constructing a resource cluster in the real field. Each resource cluster comprises a resource cluster header and multiple member vehicles. These resource cluster headers are logically connected and facilitate physical communication through member vehicles.

As the network model of RCSA, we consider a vehicular network field where numerous vehicles are positioned on roads, as illustrated in Figure 1. Each vehicle moves towards its destination based on a predetermined trajectory determined by its mobility information. Additionally, each vehicle possesses specific resources that can be shared to construct vehicular clouds. In Figure 1, we depict three types of resources: type 1, type 2, and type 3. Blue vehicles are equipped with type 1 resources, green vehicles possess type 2 resources, and red vehicles carry type 3 resources. In this paper, we define each of these vehicles as a Normal Vehicle (NV). During movement, every NV exchanges beacon messages periodically with neighboring vehicles within its communication range by broadcasting the message to share information about itself. The beacon message of an NV includes its resource types, resource amounts, position, and mobility information. Upon receiving a beacon message from a neighboring vehicle, every NV extracts the relevant information and stores it in its neighboring vehicle table. By maintaining this neighboring vehicle table, every NV can access information about all neighboring vehicles.

In this process, all NVs can establish communication with each other and form vehicular networks within the multi-hop communication range. Our scheme facilitates this communication using IEEE 802.11p Wireless Access in Vehicular Environment (WAVE) communication technology [38]. Through multi-hop communications, NVs organize themselves into resource clusters based on resource types.

In Figure 1, three resource clusters are depicted for blue vehicles, green vehicles, and red vehicles, respectively. Consequently, each resource cluster is established as a virtual entity within the real field.

### 3.2. Scheme Overview

From this point, we outline the overview of RCSA. Initially, since RCSA relies on resource clusters distinct from those utilized in existing schemes to facilitate efficient vehicular cloud construction, we delineate the process for constructing Resource Clusters (RCs) comprising vehicles with identical resource types.

In RCSA, all NVs exchange their individual information with one another and store it within their respective tables through beacon message broadcasting. Subsequently, they engage in communication and connection to establish vehicular networks irrespective of their resource types. Each NV then searches other NVs possessing the same resource type as itself using single/multi-hop communications. Following the resource type search, a Resource Cluster (RC) is formed among NVs sharing identical resource types. The NVs transition into Resource Cluster Member vehicles (RCMs) within the RC. The RCM that remains within the RC range for the longest duration is appointed as the Resource Cluster Header (RCH) for the RC. Through this process, each RC comprising solely RCMs with identical resource types can be established on the real vehicular field, where numerous vehicles with varied resource types are intermingled. For instance, in a vehicular network field featuring three resource types, each of the three RCs is conceptually established on the vehicular network field as virtual clusters 1, 2, and 3, as depicted in Figure 1. The RCH of each RC engages in periodic communication with its RCMs to oversee and sustain the RC, while also facilitating resource allocation functions to construct vehicular clouds for requester vehicles. In RCAS, the resource search and allocation process are delineated into two scenarios: intra-resource search and inter-resource search. In the case of intra-resource search, a requester vehicle seeks a resource for its vehicular cloud through a neighboring RCM affiliated with the RC possessing the same resource type as the sought-after resource. To initiate this process, the requester vehicle forwards a resource request to the neighboring RCM. Upon receiving the request, the neighboring RCM relays the request information to the RCH of the RC. Subsequently, the RCH identifies its RCMs with available resources based on the requested information and directs the selected RCMs to allocate the resources for the requester vehicle. Conversely, in the scenario of inter-resource search, a requester vehicle hunts for a resource for its vehicular cloud via a neighboring RCM associated with the RC featuring a different resource type from the desired resource. To do this, the requester vehicle submits a resource request to the neighboring RCM with a distinct resource type. Upon receiving the request, the neighboring RCM verifies if any RCM within its vicinity possesses the requested resource type. If such an RCM is found, the neighboring RCM relays the requested resource information to the RCM, which then forwards it to its RC to initiate the intra-resource search process. However, if no RCM with the requested resource type is detected, the neighboring RCM increments the hop count for resource searching and repeats the process to locate an RCM with the desired resource type. Subsequently, we outline the process for maintaining an RC. Given the dynamic nature of vehicular movement, vehicles continuously join and depart from an RC, necessitating ongoing maintenance to facilitate resource search and allocation for vehicular clouds. RC maintenance encompasses the replacement of both RCH and RCMs due to the departure of existing members from the RC. RCH replacement is crucial as it solely manages information on all RCMs within the RC. In the event of an RCH departure without a suitable replacement candidate, the RC faces dissolution. Thus, prompt RCH replacement is imperative, with the new RCH elected through the same procedure as the initial RCH. Additionally, RCM replacement occurs when a departing RCM notifies the RC of its exit and requests the retrieval of its resources. The RCH updates the RC information accordingly and recalculates the available resources. Conversely, when a new NV desires to join an RC, it forwards its resource information to the RC’s RCH, subsequently becoming a new RCM. The RCH updates the RC information with the new RCM’s details and recalculates the total available resources within the RC.

## 4. The Proposed Scheme (RCSA)

In this section, we describe the proposed scheme named Resource Cluster-based Resource Search and Allocation (RCSA) in the following three subsections. First, Section 4.1 describes the construction of resource clusters. Next, Section 4.2 describes the process through which a requester vehicle requests an available resource search and receives an allocation of a resource cluster for a vehicular cloud service. Finally, Section 4.3 describes the process of maintaining resource clusters.

### 4.1. Construction of Resource Clusters

In this section, we present the process of the construction of Resource Clusters (RCs) in RCSA. As shown in Figure 1, NVs are located on the real field and move toward their destinations. They can have resources according to their vehicle equipment capability for constructing vehicular clouds and connect for exchanging information about the resources by V2V communications based on IEEE WAVE communication technology. NVs with the same resource type are virtually grouped as an RC on the real field. Then, the RC consists of a Resource Cluster Header (RCH) and Resource Cluster Member vehicles (RCMs). To do this, we provide two phases: (1) resource type-based clustering and (2) election of an RCH. The resource type-based clustering explains the way to construct an RC by grouping NVs with the same resource type. The election of an RCH explains the way to select an RCH among all RCMs participating in an RC. We describe the resource type-based clustering and the election of an RCH in the next two subsections, respectively. Algorithm 1 shows the process of the construction of resource clusters in RCSA.

To explain Algorithm 1, we define a neighboring vehicle as $NV_{j}$, and the requester vehicle that wants to search the same type of resource as $NV_{i}$. In lines 1 to 3, the algorithm begins by updating the neighbor table that each vehicle has in the network. When $NV_{i}$ searches the same type of resource which it has and it is not equal to the type of $NV_{j}$, the process is terminated, and no further actions are taken with this neighbor. In lines 5 to 8, if both $NV_{j}$ and $NV_{i}$ have CHs, then the algorithm proceeds to check if their CHs are the same. If the CHs of $NV_{j}$ and $NV_{i}$ are the same, they are already part of the same cluster, and the process is terminated without any further action. In lines 9 to 12, if the CHs of $NV_{j}$ and $NV_{i}$ are different, the algorithm performs a comparison between them to determine which one should be the representative CH. After the comparison, the clusters represented by the two CHs are merged using the cluster merging process. In lines 13 to 16, if $NV_{j}$ is not a CH but $NV_{i}$ is a CH, $NV_{i}$ sends a *Cluster Head* message to $NV_{j}$. This is performed to request $NV_{j}$ to join the cluster. In lines 17 to 20, if $NV_{j}$ is a CH and $NV_{i}$ is not a CH, $NV_{i}$ sends a *Join* message to $NV_{j}$. This is performed to request $NV_{i}$’s joining the cluster represented by $NV_{j}$. In lines 21 to 23, regardless of whether $NV_{i}$ is a CH or not, the algorithm proceeds to compare $NV_{i}$ with $NV_{j}$ using the Comparing function. Based on the comparison results and messages exchanged, a new cluster is created, and $NV_{i}$ becomes the CH. Finally, $NV_{i}$ sends a *Cluster Head* message to $NV_{j}$.
**Algorithm 1** Resource cluster construction**Input:** Beacon msg (ID, mobility, type, amount) **Output:** Creation of resource cluster **NeighborTable.Update** (ID, mobility, type, amount)
  1:NeighborTable.Update  2:**if** $type\neq type\_NV_{i}$ **then**  3:    END  4:**end if**  5:**if** $NV_{j}$.b_CH() == 1 && $NV_{i}$.b_CH() == 1 **then**  6:    **if** $NV_{j}$.CH ≠$NV_{i}$.CH **then**  7:        END  8:    **end if**  9:    CH → Comparing($NV_{j}$.CH, $NV_{i}$.CH) 10:    Cluster Merging (CH) 11:    END 12:**end if** 13:**if** $NV_{j}$.b_CH() == 0 && $NV_{i}$.b_CH() == 1 **then** 14:    Sending CH msg to $NV_{j}$ 15:    END 16:**end if** 17:**if** $NV_{j}$.b_CH() == 1 && $NV_{i}$.b_CH() == 0 **then** 18:    Sending Join msg to $NV_{j}$ 19:    END 20:**end if** 21:CH → Comparing($NV_{j}$, $NV_{i}$) 22:Creating Cluster 23:Sending CH msg to $NV_{j}$


#### 4.1.1. Resource Type-Based Clustering

The process of creating an RC by a resource type proceeds on the vehicular network where all NVs communicate with each other periodically. All NVs are traveling along their respective routes to their destinations on the real vehicular field. Every NV has a resource with a resource type and periodically broadcasts the information on the resource type within its communication range through a beacon message. It stores the information on the resource type from all of its neighboring NVs by receiving their beacon messages in its neighboring tables. After every NV has finished storing the resource information about its neighboring NVs, it conducts resource sharing with only its neighboring NVs that have the same resource type as its resource. NVs with the same resource type are connected to create an RC for the resource type. For example, as shown in Figure 1, blue NVs have a resource type 1 and create an RC1 on the virtual field 1, orange NVs have a resource type 2 and create an RC2 on the virtual field 2, and green NVs have a resource type 3 and create an RC3 on the virtual field.

The process for constructing an RC is explained in detail as follows. Searching NVs that have the same type of resource is processed based on the range of single/multi-hop communication. First, we explain the process of searching for NVs that have the same type of resource in a single-hop communication range. NVs that have configured the network in the real field know each other for the types of resources held by neighboring NVs located within their communication range. Every NV requests each of the other NVs to share resources if any of its neighboring NVs has the same resource type as itself. When all NVs have completed resource sharing with their neighboring NVs with the same type of resource, each of them checks that it is connected to its neighboring NVs of the same resource type. Due to the mobility of vehicles, the probability that all NVs with the same type of resource are connected might be low. Thus, the next step is to expand the communication range from single-hop to multi-hop for searching NVs with the same resource type. The process of connecting NVs with the same resource type in the multi-hop range is as follows. In the single-hop communication range, some NVs may not find a neighboring NV that has the same type of resource. In this case, they only travel without searching for any NV with the same type of resource. However, since the NVs configure a vehicular network regardless of resource type, any neighboring NVs with different types of resources can exist within their communication range. In this communication range of any neighboring NVs, neighboring NVs having the same type of resource may exist. To share resources with these neighboring NVs located in the multi-hop range, they can use neighboring NVs with different types of resources as the relay vehicles to share resources with the NVs located in the multi-hop range. The single/multi-hop communications allow for the configuration of RCs between NVs with the same type of resources on the vehicular network configured in the real field.

#### 4.1.2. Election of Resource Cluster Headers

In RCSA, every RCH has the responsibility for maintaining the information of RCMs as well as joining new RCMs and leaving existing RCMs to ensure stable connectivity. To conduct this responsibility, the RCH sends and receives information from these RCMs continuously. Accordingly, the RCH needs to have a long and stable connection time with each RCM. Thus, we use the probability of connecting each vehicle in an RC as the factor to select its RCH and calculate the connection probability. In urban scenarios, roads are typically composed of two- or multi-directional roads, except for specific environments. Thus, the connection time between vehicles traveling on a two-directional road is first calculated, followed by the connection time for the multi-directional road of an intersection [39].

The connection probability between two vehicles at a specific time $t_{n}$ on a two-directional road can be obtained by calculating the location probability of the vehicles at that time. To determine this probability, the time step Δt between two consecutive time units $t_{n-1}$ and $t_{n}$ is set as the unit time, with $\Delta t=t_{n}-t_{n-1}$ and *m* representing the number of unit times. For the user’s convenience, the acceleration of autonomous vehicles is usually restricted to [−5, 5] m/s^2^, while roads typically have a legal speed limit [40,41]. At a given time $t_{n}$, the velocity of a vehicle is assumed to follow a random distribution, where the vehicles move uniformly and the acceleration experiences white Gaussian noise with a constant variance ε during the unit time Δt [42]. The velocity component of the *x*-axis for a vehicle *i* at the *k*-th unit step between two consecutive time units $t_{n-1}$ and $t_{n}$ is denoted as $v_{x}^{\left(k\right)}$, and the location component of the *x*-axis is represented by $x^{\left(k\right)}$ as follows:

(1) $$\begin{array}{cc} v_{x}^{\left(k\right)} & =v_{x}^{\left(0\right)}+\sum_{i=1}^{k}\varepsilon_{x}^{\left(i\right)}\Delta t, \end{array}$$

(2) $$\begin{array}{cc} x^{\left(k\right)} & =x^{\left(0\right)}+k\Delta tv_{x}^{\left(k\right)}+\frac{1}{2}\sum_{i=1}^{k}\left(2k-2i+1\right)\varepsilon_{x}^{\left(i\right)}\Delta t^{2}, \end{array}$$

where $\varepsilon_{x}^{\left(k\right)}$ is the acceleration component mentioned in [43]. The Gaussian distribution at $t_{n}$ is obtained as follows:

(3) $$\begin{array}{cc} \mu_{x} & =x_{n-1}+v_{\left(n-1\right)}^{x}m\Delta t, \end{array}$$

(4) $$\begin{array}{cc} \sigma_{x}^{2} & =\frac{\left(4m^{3}-m\right)\xi \Delta t^{4}}{12}. \end{array}$$

The location at the time $t_{n-1}$ is denoted by $\left(x_{n-1},y_{n-1}\right)$, and the results for the *y*-axis and *z*-axis are analogous to those of the *x*-axis. Thus, using the location $\left(x_{n-1},y_{n-1}\right)$ at $t_{n-1}$, we can obtain the probability density function for reaching $\left(x_{n},y_{n}\right)$ as follows:

(5) $$\begin{array}{cc} \; & p\{\left(x_{n},y_{n}\right)|\left(x_{n-1},y_{n-1}\right)\}\\ \; & =\frac{1}{2\pi \sigma^{2}}\exp^{\left(-\frac{1}{2\sigma^{2}}\left[{\left(x_{n}-\mu_{x}\right)}^{2}+{\left(y_{n}-\mu_{y}\right)}^{2}\right]\right)}. \end{array}$$

By considering the probability, the Gaussian distribution at the time $t_{n-1}$ can be adjusted for the distance between a vehicle α and a vehicle β as follows:

(6) $$\begin{array}{cc} \; & \mu_{d}\left(\alpha ,\beta \right)={\left(\mu_{x}^{\alpha }-mu_{x}^{\beta }\right)}^{2}+{\left(\mu_{y}^{\alpha }-\mu_{y}^{\beta }\right)}^{2}, \end{array}$$

(7) $$\begin{array}{cc} \; & \sigma_{d}^{2}\left(\alpha ,\beta \right)\\ \; & =\frac{\Delta t^{4}}{4}\\ \; & \times E\left[\sum_{i=1}^{m}{\left(2i-1\right)}^{4}\left({\left(\varepsilon_{\alpha ,x}^{(k)2}-\varepsilon_{\beta ,x}^{(k)2}\right)}^{2}+{\left(\varepsilon_{\alpha ,y}^{(k)2}-\varepsilon_{\beta ,y}^{(k)2}\right)}^{2}\right)\right], \end{array}$$

The Gaussian distribution for the vehicle α is denoted by $\mu_{x}^{\alpha }$, $\mu_{y}^{\alpha }$, $\varepsilon^{\left(k\right)}\alpha ,x$, and $\varepsilon^{\left(k\right)}\alpha ,y$, while the Gaussian distribution for the vehicle β is represented by $\mu_{x}^{\beta }$, $\mu_{y}^{\beta }$, $\varepsilon^{\left(k\right)}\beta ,x$, and $\varepsilon^{\left(k\right)}\beta ,y$. Therefore, the probability density function for reaching $d_{\gamma }$ is obtained based on the distance $d_{n-1}$ between the vehicle α and the vehicle β at time $t_{n-1}$ as follows:

(8) $$\begin{array}{cc} p\{d_{n}\left(\alpha ,\beta \right) & =d_{\gamma }\left|d_{n-1}\left(\alpha ,\beta \right)\right\}\\ \; & =\frac{1}{2\pi \sigma^{2}}\exp^{\left(-\frac{1}{2\sigma^{2}}\left[{\left(d_{n}-\mu_{d}\left(\alpha ,\beta \right)\right)}^{2}\right]\right)}. \end{array}$$

The probability of the distance between two vehicles being within the communication range $R_{V2V}$ of vehicle-to-vehicle at time $t_{n}$ can be determined by integrating under $d_{n}$, and it can be calculated as follows:

(9) $$Pr_{conn}^{\left[\alpha ,\beta \right]}\left(t_{n}\right)=\int_{0}^{R_{V2V}}p\left\{d_{n}\left(\alpha ,\beta \right)=q|d_{n-1}\left(\alpha ,\beta \right)\right\}\;dq.$$

Subsequently, the connection probability between two vehicles at time $t_{n}$ on a two-directional road is utilized to calculate the probability of their connection on a multi-directional road. To accurately calculate the connection probability between two vehicles at a given time $t_{n}$, it is crucial to consider the probability of each vehicle’s trajectory. A Markov model [44], which takes into account each vehicle’s historical data, can estimate the trajectory probability of the vehicle. However, the accuracy of the Markov model is affected by the complexity of the model, which increases with the order number. Thus, the 1st-order Markov model is commonly used to estimate the trajectory probability. The trajectory probability of a 1st-order Markov model is calculated as follows:

(10) $$p_{i(i+1)}=\mathrm{Pr}\left(L_{\left(i+1\right)}|L_{i}\right)=\frac{X(L_{i},L_{\left(i+1\right)})}{Z_{\left(L_{i}\right)}},$$

where $p_{i(i+1)}$ is the trajectory probability that is derived based on the number of times that the vehicle moves to the next location $L_{\left(i+1\right)}$ when it drive at a current location $L_{i}$. $X(L_{i},L_{\left(i+1\right)})$ represents the number of transitions observed from state $L_{i}$ to the state $L_{\left(i+1\right)}$, and $Z(L_{i})$ represents the number of transitions observed from the state $L_{i}$ to any other states.

For an intersection that has more multiple directions, the trajectory probability is calculated using a 2nd-order Markov model based on the calculated Equation (10) using the 1st-order Markov model. Then, the trajectory probability of a 2nd-order Markov model can be expressed as follows:

(11) $$\begin{array}{cc} p_{\left(i-1)i(i+1\right)} & ={\mathrm{Pr}}_{(}L_{\left(i+1\right)}\left|L_{i},L_{\left(i-1\right)}\right) \end{array}$$

(12) $$\begin{array}{cc} \; & =\frac{X(L_{\left(i+1\right)},L_{i},L_{\left(i-1\right)})}{Z(L_{i},L_{\left(i-1\right)})}, \end{array}$$

where $p_{\left(i-1)i(i+1\right)}$ represents the probability of the vehicle’s trajectory from $L_{\left(i-1\right)}$ to $L_{i}$ and then to $L_{\left(i+1\right)}$ based on the number of times the vehicle has traveled this path. $X(L_{\left(i+1\right)},L_{i},L_{\left(i-1\right)})$ is the frequency of observing the sequence $L_{\left(i-1\right)},L_{i},L_{\left(i+1\right)}$, while $Z(L_{i},L_{\left(i-1\right)})$ is the normalization factor that ensures the sum of all possible trajectories from $L_{\left(i-1\right)}$ to $L_{i}$ and then to any next location $L_{j}$ except $L_{\left(i-1\right)}$ to $L_{i}$ is equal to one.

Depending on the order of the Markov model, the accuracy of the trajectory prediction increases but leads to a tremendous increase in complexity. Thus, we only predict the trajectory by using the 2nd-order Markov model. According to the location $\left(x_{n-1},y_{n-1}\right)$ of a vehicle α at time $t_{n-1}$, the probability of the vehicle to reach the location $\left(x_{n},y_{n}\right)$ at time $t_{n}$ while choosing the *s*-th path among *S* available paths can be represented as follows:

(13) $$\begin{array}{cc} \; & \mathrm{Pr}[\alpha_{n}^{s}\left(\left(x_{n},y_{n}\right)\right)]\\ \; & =p\left\{\left(x_{n},y_{n}\right)|\left(x_{n-1},y_{n-1}\right)\right\}\times p_{(n-1)n,s}^{\alpha }. \end{array}$$

Then, to calculate the expected connection probability between two vehicles α and β at the time $t_{n}$, we define the cumulative distribution function (CDF) by considering the trajectory probability of each of them as follows:

(14) $$P_{\mathrm{conn},\alpha \beta }\left(t_{n}\right)=\underset{D}{\;\int \int \;}P\left(d_{\alpha \beta },s|t_{n}\right)f\left(d_{\alpha \beta }\right)dsdd_{\alpha \beta },$$

where *D* represents the possible values of $d_{\alpha \beta }$ and *s* for a given time $t_{n}$, $f(d_{\alpha \beta })$ is the probability density function of the distance $d_{\alpha \beta }$ between the two vehicles α and β, and $P(d_{\alpha \beta },s|t_{n})$ is the conditional probability that the two vehicles are connected when they are at a distance $d_{\alpha \beta }$ and follow the *s*-th path at the time $t_{n}$. Based on this information, the overall probability of the connection between the vehicle α and the vehicle β at the time $t_{n}$ can be calculated using the product of the conditional connection probabilities between each pair of two consecutive locations $L_{i}$ and $L_{\left(i+1\right)}$ along their respective trajectories. Therefore, the connection probability between the vehicles α and β at the time $t_{n}$ can be represented as follows:

(15) $$Pr_{conn}^{\alpha ,\beta }\left(t_{n}\right)=\prod_{q=1}^{Q^{\alpha ,\beta }}Pr_{conn}^{i,(i+1)}\left(t_{n}\right).$$

After calculating the connection probabilities between vehicles using Equation (14), each vehicle in an RC determines its connection time with its neighboring vehicles. The average connection time with neighbors is then calculated, and the vehicle with the highest average connection time is chosen as the RCH in the RC. This selection is based on the ability to maintain a stable connection with neighboring vehicles for the longest time in its RC, thereby facilitating our RC management. By this RCH election process, the selected RCH in an RC sends an RCH_Selection message with its ID and location information to all RCMs in the RC to inform them of its selection. On receiving the RCH_Selection message, every RCM in the RC recognizes the ID of the selected RCH and sends an *Acknowledge* message with its available resource amount information to the selected RCH. By gathering *Acknowledge* messages from all RCMs in the RC, the selected RCH knows the information of their IDs and their available resource amounts and saves the information in an RCM table. As a result, vehicles with the same resource type construct an RC consisting of an RCH and RCMs. Since it is also very important to know the latest information about RCMs and their resource amounts in each RC for effectively constructing vehicular clouds, every RCM periodically sends an *Update* message with its ID, location, and available resource amount to its RCH.

### 4.2. Resource Search and Allocation

This section presents the process for searching for the desired type of resource and for allocating the resource to construct a VC through using RCs. This process is divided into two schemes: intra-resource search, and inter-resource search. The intra-resource search means that a requester vehicle as an NV searches RCMs for allocating the required amount of resources in its RC with the same resource type as itself to construct a VC. On the other hand, the inter-resource search means that a requester vehicle as an NV searches RCMs for allocating the required amount of resources in another RC with a different resource type from itself to construct a VC. We describe the intra-resource search and the inter-resource search in detail in the next two subsections, respectively.

Algorithm 2 shows the process of resource search and allocation in RCSA. To explain Algorithm 2, we define a *q* as the neighboring vehicle of the requester vehicle within its communication range. Candidate is the neighboring vehicle that has the same type of resource as the requester vehicle. maxConn is the maximum connection time between the candidate and the requester vehicle.
**Algorithm 2 ** Resource search and allocation$V_{req}$ is *i* **Input:** Request message ($ID_{i}$, $pos_{i}$, $vel_{i}$, $type_{i}$, $res_{i}$) **Output:** next ID
  1:next == NULL  2:**for** *q*∈neighborTable **do**  3:    **if** ($type_{q}$ == $type_{i}$) **then**  4:        **if** (q.isCH()) **then**  5:           **return** *q*  6:        **else**  7:           Candidate[1] ←*q*  8:        **end if**  9:    **else** 10:        **if** ($q.isNeighborTypes(type_{i})$ != 0) **then** 11:           Candidate[2] ←*q* 12:        **else** 13:           **if** (q.isCH()) **then** 14:               Candidate[3] ←*q* 15:           **else** 16:               **if** (q.ifNeighborOfCH) **then** 17:                   Candidate[4] ←*q* 18:               **else** 19:                   Candidate[5] ←*q* 20:               **end if** 21:           **end if** 22:        **end if** 23:    **end if** 24:**end for** 25:**for** *p* = 1 to 5 **do** 26:    **if** (Candidate[i].isEmpty()) **then** 27:        next ←Candidate[i].maxConn() 28:        **return** next 29:    **end if** 30:**end for**


Enter the neighbor into a candidate set with different priorities according to Algorithm 2. First, determine if the neighbor’s resource type is the same as the requested resource type. If both types are the same and the neighbor is a CH, it immediately requests to join the resource cluster. If the neighbor is not a CH, it is entered into the candidate set with the priority because it has the information of a CH. If the neighbor’s resource type is different from the requested resource type, two more things are checked. First, check if there are any vehicles in the neighbors that belong to the requested resource type. If this neighbor has a vehicle with the requested resource type in its communication range, it is entered into the second priority candidate. If this neighbor is a CH with a different type of resource, this vehicle is entered in the third priority candidate set because it can access the CH with the requested resource type through CH-to-CH communication. In addition, this neighbor checks if any vehicles are CHs. If there are vehicles in the neighbors that are CHs, the neighbor is entered into the fourth priority candidate set. If not, the neighbor is entered into the lowest priority candidate set with the lowest rank.

After the candidate set input is complete, check at the highest priority candidate set first. If the high-priority candidate set is empty, check the next priority candidate set. If the candidate set is not empty, the vehicle selects the vehicle with the longest connection time from that candidate set and selects it as the next node to forward the packet.

#### 4.2.1. Intra-Resource Search

**Figure 2:**
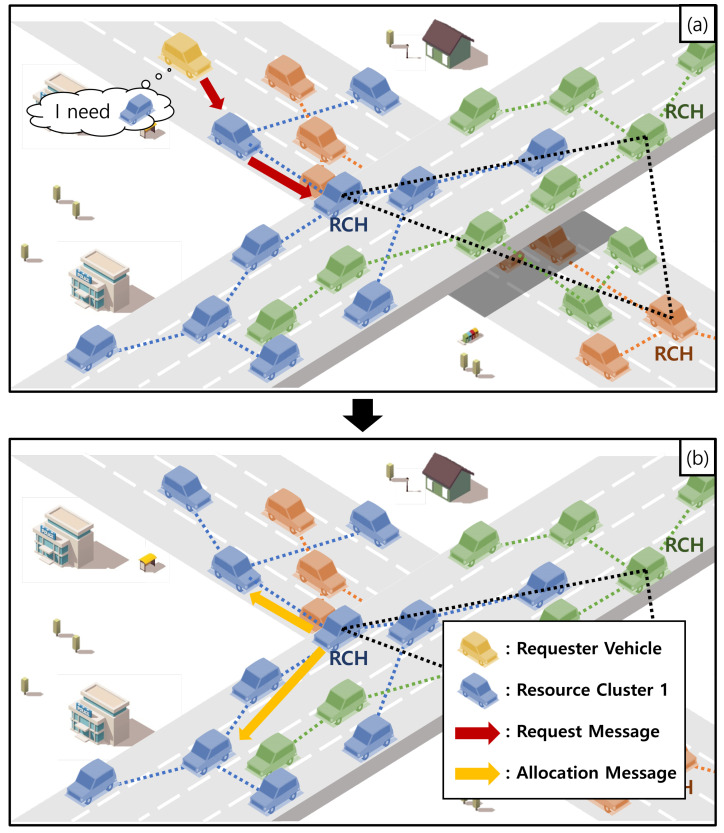
The intra-resource search and allocation process: (**a**) resource request from a requester vehicle to an RCH; (**b**) resource allocation from an RCH to RCMs.

In this section, we present the intra-resource search. Figure 2 shows an example of the intra-resource search. In the network model of this paper, any vehicle can construct a vehicular cloud to exploit a vehicular cloud service in a vehicular network field. This vehicle is defined as a requester vehicle in this paper. In our resource cluster-based network architecture, there can be two types of requester vehicles. The first type is requester vehicles as NVs. As shown in Figure 2, if a vehicle (i.e., the yellow vehicle) as an NV wants to use a vehicular cloud service with a resource type on moving toward its destination, it becomes a requester vehicle $V_{req}$ as the type of NV for the vehicular cloud. In this case, to construct a vehicular cloud with the resource type, $V_{req}$ needs to find the RC with the resource type because it should be allocated the resource from the RCH of the RC. To do this, $V_{req}$ checks whether anyone among its neighboring vehicles in its communication range is an RCM (i.e., a blue vehicle) in the RC by its neighboring vehicle table. If $V_{req}$ has this RCM as its neighboring vehicle, it requests the resource to construct the vehicular cloud to the RC by using the RCM. For requesting the resources to construct the vehicular cloud, $V_{req}$ sends a *Request* message with the information of the resource type and the required resource amount to the RCM to require the resource to the RC as shown in Figure 2a. On receiving the *Request* message from $V_{req}$, the RCM checks whether the requested resource type in the *Request* message is the same as its resource type. Because its resource is the same as the requested resource type, the intra-resource search process is conducted. To further relay the *Request* message to the RCH (i.e., the blue RCH), the RCM sends the message to the next RCM of the RC toward the RCH. On receiving the *Request* message, the next RCM performs the same process as the previous RCM that sent the message to it. By this process, the RCH of the RC can consequently receive the *Request* message of $V_{req}$ through relaying the message by RCMs because every RCM knows the route to the RCH.

There can be the second type of requester vehicle as an RCM in this paper. If an RCM in an RC wants to construct a vehicular cloud with its resource (that is the resource type in the RC), it becomes a requester vehicle $V_{req}$ as the type of RCM for the vehicular cloud. In this case, since $V_{req}$ is an RCM in the RC, the intra-resource search process is conducted. For constructing the vehicular cloud, $V_{req}$ as the RCM sends a *Request* message with the information of the resource type and the required resource amount to the next RCM of the RC toward the RCH. As in the NV type, the *Request* message is eventually sent to the RCH through relaying the message by RCMs If the RCH receives a request message from a requester vehicle $V_{req}$ as an NV or an RCM, it selects RCMs that can provide the requested resource amount in the message among RCMs in the RC. When selecting RCMs for the requested resource amount, the RCH can have multiple candidate combinations of RCMs.

The RCH chooses the optimal combination that can provide the requested resource amount to $V_{req}$ as a minimum number of RCMs and a high connection time among vehicles. Since multiple requester vehicles can request the same resource from the RCH in our network model, the minimum RCMs should be selected to meet the requirements of requester vehicles as much as possible. If RCMs to allocate resources are selected, the RCH sends an *Allocation* message to each of the RCMs as shown in Figure 2b. Algorithm 3 shows the summary of the intra-resource search process. We describe how to select RCMs for resource allocation in Section 4.2.3.
**Algorithm 3** Intra-Resource Search$V_{req}$ is *i* **Input:** Request message ($ID_{i}$, $pos_{i}$, $vel_{i}$, $type_{i}$, $res_{i}$) **Output:** next ID
  1:next == NULL  2:**for** *q*∈neighborTable **do**  3:    **if** ($type_{q}$ == $type_{i}$) **then**  4:        **if** (q.isCH()) **then**  5:           **return** *q*  6:        **else**  7:           Candidate[0] ←*q*  8:        **end if**  9:    **end if** 10:**end for** 11:**return** Candidate[i].maxConn()


#### 4.2.2. Inter-Resource Search

In the inter-resource search process, two types of requester vehicles aim to construct vehicular clouds: Normal Vehicles (NVs) and Resource Cluster Members (RCMs). NVs are vehicles searching for resources to construct vehicular clouds, while RCMs are already part of resource clusters and are capable of providing resources to NVs searching to construct vehicular clouds.

**Figure 3:**
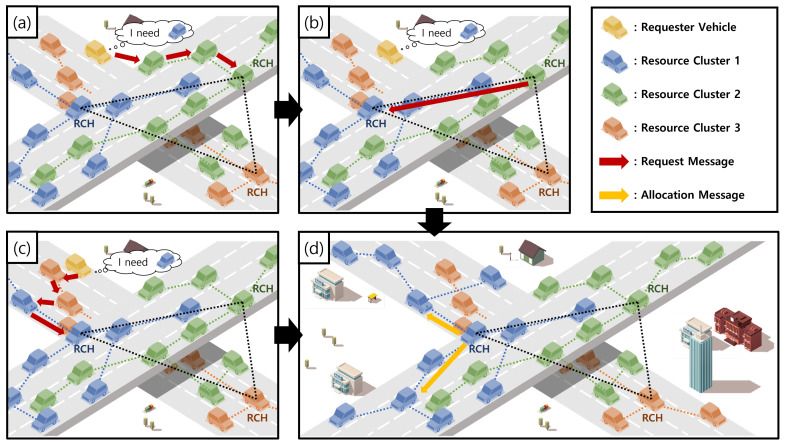
The resource search in the inter-resource: case (1), RCHs are related in searching as in (**a**)→(**b**)→(**d**); case (2), only members are related in searching as in (**c**) →(**d**).

Figure 3 shows an example of the inter-resource search. As the first type, a vehicle as an NV becomes a requester vehicle $V_{req}$ when it wants to construct a vehicular cloud with a resource type. For constructing the vehicular cloud by being allocated the resource, $V_{req}$ should find the RC of the resource type. However, when $V_{req}$ checks its neighboring vehicles in its communication range, it cannot have any RCMs of the RC as a neighboring vehicle different from the intra-resource search. Then, for requesting the resource, $V_{req}$ arbitrarily selects one of its neighboring vehicles which is an RCM of any RC different from the RC of the requested resource type. For constructing the vehicular cloud, $V_{req}$ sends a *Request* message with the information of the resource type and the required resource amount to the RCM as shown in Figure 3a. On receiving the *Request* message from $V_{req}$, the RCM checks whether the requested resource type in the *Request* message is the same as its resource type. Because its resource type is different from the requested resource type, the inter-resource search process is conducted. In the proposed scheme, the inter-resource search can be divided into two cases because vehicles maintain their neighbor tables. The first case is shown in Figure 3a,b,d. In the first case, the RCM (i.e., the green RCM) further sends the *Request* message to the RCH (i.e., the green RCH) of its RC by relaying other RCMs (i.e., other green RCMs) as shown in Figure 3a. In this case, all RCMs that participated in relaying the *Request* message do not have any RCM (i.e., a blue RCM) of the requested resource type in their own neighbor table. On receiving the *Request* message, the RCH (i.e., the green RCH) also sends the *Request* message to the RCH (i.e., the blue RCH) of the requested resource type in the *Request* message as shown in Figure 3b. If the RCH of the requested resource type finally receives the *Request* message of $V_{req}$, it selects RCMs that can provide the requested resource amount in the message among RCMs in its RC as shown in Figure 3d. The second case is shown in Figure 3c,d. Sometimes, any RCM (i.e., a red RCM in Figure 3c that participated in relaying the *Request* message can have any RCM (i.e., the blue RCM) of the requested resource type in the *Request* message in its neighbor table different from the first case. Then, the RCM with the RCM of the requested resource type sends the *Request* message to the RCM of the requested resource type as shown in Figure 3c. On receiving the *Request* message, the RCM of the requested resource type further sends the *Request* message to its RCH (i.e., the blue RCH). In the first case, the RCH of the requested resource type finally receives the *Request* message and selects RCMs in its RC for the requested resource amount of $V_{req}$ as shown in Figure 3d.

In the intra-resource search, there can be a second type of requester vehicle as RCMs in the inter-resource search. If an RCM in an RC wants to construct a vehicular cloud with a resource type that is different from its resource type, it becomes a requester vehicle $V_{req}$ as the RCM. In other words, the RCM as $V_{req}$ does not belong to the RC of the requested resource type. If the RCM does not have any RCMs in the RC of the requested resource type as a neighboring vehicle in its neighbor table, the second type of requester vehicles case also conducts the inter-resource search process. The second type can also have two cases in the inter-resource search process according to the information of vehicles in the neighbor tables like the requester vehicles as NVs. For constructing the vehicular cloud in the first case, the RCM as $V_{req}$ sends a *Request* message with the information of the resource type and the required resource amount to the RCH of its RC by relaying other RCMs because all RCMs that participate in relaying the *Request* message do not have any RCMs of the requested resource type in their neighbor table. If the RCH receives the *Request* message, it further sends the *Request* message to the RCH of the requested resource type in the *Request* message. Finally, the RCH of the requested resource type receives the *Request* message and selects RCMs in its RC for the requested resource of $V_{req}$ for resource allocation. For the second case different from the first case, any RCM that participates in relaying the *Request* message can have any RCM of the requested resource type in the *Request* message in its neighbor table. Then, the RCM with the RCM of the requested resource type sends the *Request* message to the RCM of the requested resource. The RCM of the requested resource further sends the *Request* message to its RCH. On receiving the *Request* message, the RCH selects RCMs in its RC for the requested resource of $V_{req}$ for resource allocation. Algorithm 4 shows the summary of the inter-resource search process. As the intra-resource search, we describe how to select RCMs for the resource allocation in Section 4.2.3.
**Algorithm 4** Inter-Resource Search$V_{req}$ is *i* **Input:** Request message ($ID_{i}$, $pos_{i}$, $vel_{i}$, $type_{i}$, $res_{i}$) **Output:** next ID
  1:next == NULL  2:**for** *q*∈neighborTable **do**  3:    **if** ($type_{q}$≠$type_{i}$) **then**  4:        **if** ($q.isNeighborTypes(type_{i})$ != 0) **then**  5:           Candidate[0] ←neighbor  6:        **else**  7:           **if** (q.isCH()) **then**  8:               Candidate[1] ←neighbor  9:           **else** 10:               **if** (q.ifNeighborOfCH) **then** 11:                   Candidate[2] ←neighbor 12:               **else** 13:                   Candidate[3] ←neighbor 14:               **end if** 15:           **end if** 16:        **end if** 17:    **end if** 18:**end for** 19:**for** *p* = 0 to 3 **do** 20:    **if** (Candidate[i].isEmpty()) **then** 21:        next ←Candidate[i].maxConn() 22:        **return** next 23:    **end if** 24:**end for**


#### 4.2.3. Resource Allocation

For constructing a VC of a resource type, a requester vehicle $V_{req}$ needs to be allocated the resources from the RCMs of the RC for the resource type through intra-resource or inter-resource searches. Then, $V_{req}$ requests the resource allocation to the RCH of the RC. When $V_{req}$ uses the intra-resource search, if there is an RCM belonging to the RC of the same resource within the communication range of $V_{req}$, $V_{req}$ sends the information about the required amount of the resource to the RCM. Upon receiving the information, the RCM further sends it to the next RCM toward its RCH. Through this process, the information received from $V_{req}$ is finally transmitted to the RCH. Then, the RCH determines whether the required amount of the resource of $V_{req}$ can be fully allocated by RCMs in its RC. If the RC has a sufficient amount to allocate the required amount of $V_{req}$, the RCH selects the RCMs that have high connection times with $V_{req}$ and available resource amounts among all RCMs. The RCH requests the selected RCMs to allocate their available resources for $V_{req}$ and sends the resource and mobility information of the selected RCM to $V_{req}$. Accordingly, $V_{req}$ can leverage the resources of the selected RCMs to form a VC through single or multiple communications with them and use the service of the VC. On the other hand, when $V_{req}$ uses the inter-resource search, $V_{req}$ requests the required amount of a resource type to an RCM of RCs with different resource types within its communication range. As the process in the intra-resource search, the request of $V_{req}$ is transmitted to the RC of the RCM with a different resource type from the required resource. Since the RCH has a different resource type, it further forwards the request to the RCH of the required resource type from $V_{req}$. On receiving the request, the RCH of the required resource type performs the same resource allocation process to select RCMs to allocate the required amount as in the intra-resource search.

To explain the resource allocation process in more detail, we present an example of resource allocation. Based on YouTube recommendations, the length of the common 1080p video is 3 to 15 min and its size is 150 MB to 1 GB [45].

**Figure 4:**
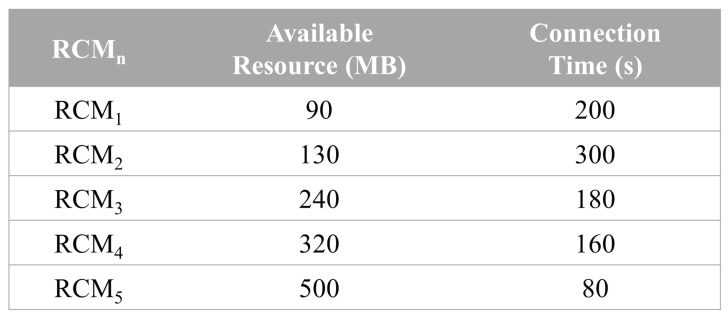
An example of an RCM table for $V_{req}$ in the RCH of the storage resource type. Each element has the ID of an RCM, its available resource amount, and its connection time with $V_{req}$.

For example, let $V_{req}$ download a video from YouTube, which is 500 MB in size and 600 s in length. If $V_{req}$ only has the storage resource of 100 MB to save the video, it cannot download the whole of the video by itself. Thus, $V_{req}$ needs to request the lack amount of 400 MB of the resource for 600 s to the RCH of the resource type. To do this, it sends a *Request* message with the required information of 400 MB and 600 s to the RCH by relaying the message to RCMs of the RCH. On receiving the *Request* message, the RCH selects the RCMs that can satisfy the required information from $V_{req}$ among all RCMs in its RCM table as shown in Figure 4. Based on the RCM table, RCMs that can satisfy the requirement of $V_{req}$ are a set of RCMs 1, 2, and 3 (460 MB, 680 s) and a set of RCMs 2, 3, and 4 (690 MB, 640 s). In RCSA, among them, we select the set that uses the least amount of the resource among all available sets to satisfy the requirement of $V_{req}$. Thus, the set of RCMs 1, 2, and 3 are selected to allocate their resource for $V_{req}$. Since using more resource amount than the requirement of $V_{req}$ may result in resource waste for the RCMs providing the resource when any of them becomes a new $V_{req}$, it will not receive the resource back. For the resource allocation to $V_{req}$, the RCH sends an *Allocation* message to each of the RCMs in the selected set. On receiving the *Allocation* message from the RCH, the RCMs allocate their resource for $V_{req}$.

### 4.3. Maintenance of Resource Clusters

In this section, we address the maintenance of RCs in RCSA. The maintenance of RCs is divided into two processes. The first process is replacing the existing RCH with a new RCH in an RC because the existing RCH can leave the RC due to its mobility. The second process is managing RCMs in an RC because any vehicle can join as an RCM in the RC or any RCM can leave the RC. We describe the two processes, the replacement of RCHs and the management of RCMs, in the next two subsections, respectively.

#### 4.3.1. Replacement of Resource Cluster Headers

The leaving of the RCH in an RC is a critical issue that destructs the RC. The RCH has an important role in periodically managing the information of all RCMs within the RC and is responsible for the resource search and allocation according to the requirement from $V_{req}$. If an RCH leaves its RC by changing its mobility or by using the RC for making a VC, it must be quickly replaced by a new RC through the election of the RCH. As a new RCH, the existing RCH selects the RCM with the highest score among all RCMs according to the equation presented for the election of resource cluster headers in Section 4.1.2. After electing the new RCH, the existing RCH needs to inform all RCMs in the RC of the fact that the new RCH has been changed. To do this, the existing RCH sends an RCH_Selection message to the RCM with the highest score to inform the selection as the new RCH. On receiving the RCH_Selection message, the RCM with the highest score sends an *Acknowledge* message as the response to accept the new RCH election and eventually becomes the new RCH in the RC. If the existing RCH receives the *Acknowledge* message, it recognizes the completion of the new RCH selection and leaves the RC. To inform all RCMs in the RC of the replacement of the new RCH, the RCH sends an RCH_Change message to all RCMs in the RC. The RCH_Change message includes information about the ID and mobility of the new RCH. On receiving the RCH_Change message, each RCM saves the information about the new RCH and sends a Join message with its ID, mobility, and resource information to the new RCH. On receiving Join messages from RCMs, the new RCH stores the information about them in its RCM table. Furthermore, the new RCH informs other RCHs with different types of resources that it has become the new RCH. Accordingly, it sends another RCH_Change message to each of the other RCHs. On receiving the RCH_Change message, the other RCHs recognize the replacement of the new RCH, save the information about it, and send an *Acknowledge* message with the information of their ID and mobility as the response to the new RCH. On receiving *Acknowledge* messages from the other RCHs, the new RCH saves the information about their ID and mobility. By using the information of the other RCHs and RCMs obtained from this process, the new RCH can update and manage the information about all of them.

#### 4.3.2. Management of Resource Cluster Members

Generally, to participate in the vehicular network field, a vehicle needs to join as a new RCM in an RC based on its resource type. Any existing RCM in an RC can also leave the RC due to getting out of the vehicular network field. Accordingly, the management of joining a new RCM and leaving an existing RCM in an RC is also a critical issue for the maintenance of RCs in RCSA. As an event for the management of RCMs, when an existing RCM leaves its RC, it sends a *Leave* message to its RCH to inform it of its leaving. The *Leave* message includes the information about the ID and resources of the RCM. On receiving the *Leave* message, the RCH checks whether it allocates the resource of the RCM for making a VC or not. If the resource of the RCM is not allocated, the RCH sends an *Acknowledge* message as the response for the *Leave* message to the leaving RCM and deletes the information of the RCM in its RCM management table. However, if the RCH allocates the resource of the RCM, it needs to replace the RCM with another RCM among all RCMs in the RC. As the replacing RCM, the RCH chooses one among RCMs that have more resources than the allocated resource amount of the leaving RCM to maintain the VC continuously during its service duration. To do this, the RCH sends an *Allocation* message with the information of the allocation amount and duration of the resource to the chosen RCM and updates the information about the resource of the chosen RCM in its RCM management table. On receiving the *Allocation* message, the chosen RCM uses the allocated resource amount during the allocation duration for the VC. The RCH also sends an *Acknowledge* message as the response to the *Leave* message to the leaving RCM. Then, the RCH recalculates the available amount of the resource in the RC based on the updated information about RCMs in its RCM management table. As another event for the management of RCMs, when a vehicle comes into the vehicular network field, it wants to join for becoming an RCM in an RC according to its resource type. To do this, it gathers Beacon messages from its neighboring vehicles within its communication range. Through the information about the neighboring vehicles in the beacon messages, it checks whether some neighboring vehicle has the same resource type as its resource type or not. If it finds a neighboring vehicle with the same resource type, it requests and receives the ID and location information of the RCH of the RC for the resource type of the neighboring vehicle. With the information of the RCH, it sends a *Join* message with its ID and resource information to the RCH to become a new RCM. On receiving the *Join* message, the RCH adds the new RCM to its RCM management table, recalculates the available amount of the resource in the RC, and sends an *Acknowledge* message to the new RCM. On receiving the *Acknowledge* message, the joining of the new RCM in the RC is completed. On the other hand, if the vehicle has no neighboring vehicle with the same resource type because all of its neighboring vehicles have resource types different from its resource type, it sends a *Request* message with its ID, resource type, and location information to one of its neighboring vehicles to find out the RCH of the RC for its resource type. On receiving the *Request* message, the neighboring vehicle further relays the *Request* message to its RCH. On receiving the *Request* message, the RCH finds the RCH for the resource type in the *Request* message and sends a *Response* message with the ID and location information of the RCH of the requested resource type to the vehicle of the *Request* message. If the vehicle receives the *Response message*, it sends a *Join* message to the RCH included in the *Response* message to become a new RCM of the RCH. On receiving the *Join* message, the RCH adds the new RCM to its RCM management table, recalculates the available amount of the resource in the RC, and sends an *Acknowledge* message to the new RCM. On receiving the *Acknowledge* message, the joining of the new RCM in RC is completed.

## 5. Performance Evaluation

In this section, we compare the performance of the proposed scheme (Named RCSA) with that of the previous scheme (SERVitES) [22] through simulations. We first describe our simulation environments and performance evaluation metrics. We next evaluate the performances of the proposed scheme and the previous scheme through simulation results.

### 5.1. Simulation Environment

To evaluate the performances of SERVitES and the proposed scheme, we implemented them in a Network Simulator-3 (NS-3) version 3.34. To apply the mobility of vehicles in the city area, we included an enhanced Manhattan Mobility Model in the NS-3. In the network size of 2 km × 2 km, intersections existed at the interval of 1 km, and RSUs were deployed for each intersection. The RSUs had a communication range of up to 800 m and a transmission rate of up to 54 Mbps based on the IEEE 802.11p communication standard. The vehicles had a density of 100 vehicles per 1 km^2^ and travelled at an average speed of 40 km/h on a two-lane road. The speed of the vehicles was determined by an acceleration value according to the Gaussian distribution limited to [−5, 5] m/s^2^ every second [40,41]. The probability of selecting a driving direction at each intersection for each vehicle follows the Markov Model. Furthermore, vehicles have a communication range of up to 200 m and a transmission rate of up to 54 Mbps. The list of simulation parameters is given in Table 1.

To evaluate the proposed scheme, we compared its performance with SERVitES because they only use V2V communications to construct vehicular clouds. The proposed scheme considers various kinds of resources but SERVitES does not consider them. Unlike the proposed scheme, SERVitES divides a vehicular network into clusters called cells. Furthermore, in SERVitES, when a requester vehicle enters a cluster or leaves the cluster due to its mobility, it will search for a new cluster within a maximum of 3 hops. Due to these reasons, the performance of SERVitES is highly dependent on the cluster range and vehicle-to-vehicle connectivity.

Thus, we compared three performances of the proposed scheme with SERVitES according to four environmental values. The four environmental values were the density of vehicles, the ratio of the requester vehicles, the average speed of vehicles, and the number of the considered resource types, respectively. The density of vehicles is the number of vehicles in the 1 km × 1 km network area. If the density of vehicles is high, the requester can easily find a cluster of the requested resources. This has a direct impact on finding the cluster. The ratio of the requester vehicles reflects the popularity of the requested resource as a percentage of requester vehicles among all vehicles. The average speed of vehicles is the average of the vehicle’s speed based on the determined acceleration by the Gaussian distribution every second from the start of the simulation in the entire network area. It directly affects vehicle-to-vehicle connectivity when maintaining the RC. However, we only consider it to search the RC. In this consideration, the speed affects the new connection of other vehicles with the requester vehicles. The number of the considered resource types denotes the number of subdivided clusters to provide various kinds of resource services. It is good for compatibility but causes a lack of member vehicles in the cluster because clusters are divided according to the resource types.

The performances of the proposed scheme and SERVitES were estimated in terms of the resource searching delay, the number of packets, and the success ratio.

The resource searching delay is the time from requesting the resource by the requester vehicle to allocating the resource to the requester vehicle. If the resource searching delay is long, the requester vehicles suffer buffering and, in the worst cases, the service will be interrupted. In the proposed scheme, since all the vehicles have information about their neighbors’ resources and clustering, the time to search the vehicular cluster of the requested resource is decreased.

The number of packets is the number of transmitted packets during the resource searching delay. It includes the number of packets to search the cluster members of the requested resource, to make the requester vehicle join the vehicular cluster, and to allocate the cluster member vehicle to the requester vehicle. Furthermore, it includes the number of packets to make a new vehicle when there are no members of any vehicular cluster among neighbors of the requester vehicle in the network area. The increased number of packets can cause congestion and collision in the network and disrupt other vehicles’ various services. The proposed scheme reduces the number of packets through the information of neighbors’ resources and clustering.

The success ratio is the percentage of the number of successes in allocating the requested resources to the requester vehicle by the member vehicles of the vehicular cluster for the number of requests for resources by the requester vehicles. The requester vehicle may not receive the requested resource due to it failing to search the vehicular cluster of the requested resource. To prevent this failure, all the vehicles have neighbors’ information about resources and clustering in the proposed scheme. Furthermore, if none of the neighbors of the requested vehicle has the same type of resource as the requested resource, the neighbor with the other type of resource forwards the request message to the CH of its type or the neighbor of a neighbor with the same type of resource forwards the request message to the neighbor. The CH with the other type of resource forwards the request message to the CH with the same type of resource using the table between CHs. Therefore, the proposed scheme improves the success ratio using these forwarding algorithms.

### 5.2. Simulation Results

#### 5.2.1. The Resource Searching Delay

In RCSA, when a vehicle sends a join message to the RC requesting a specific resource type, its neighbors can efficiently forward the message to other vehicles with the same resource type. This efficient communication is enabled through the intra and inter-resource search where all vehicles periodically exchange information about their resource type and mobility. On the contrary, SERVitES constructs vehicular clusters based on vehicle mobility without considering the resource type. Consequently, a requester vehicle broadcasts the request message to its neighbors until it identifies a vehicle with a matching resource type. If no vehicle with the required resource type exists in the current cell, the requester vehicle must wait until it enters the next cell to continue the search. For this reason, the proposed scheme has fewer resource searching delays than SERVitES.

**Figure 5:**
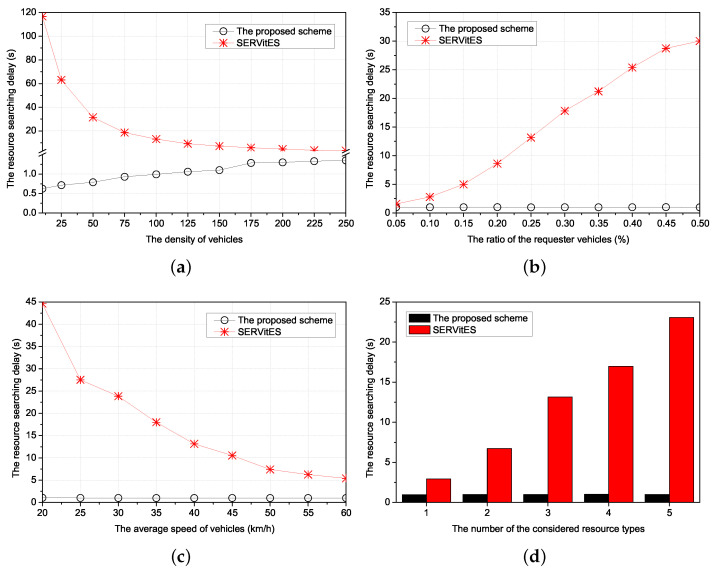
The resource searching delay according to (**a**) the density of vehicles, (**b**) the ratio of the requester vehicles, (**c**) the average speed of vehicles, and (**d**) the number of the considered resource types.

Figure 5a shows the resource searching delay according to the density of vehicles. When vehicles are sparse, the requester vehicles hardly find the RC that has the requested type of resource among their 1 hop neighbors. In SERVitES, if the requester vehicles do not have neighbors of the RC, they have to find the RC via 2 or 3 hops neighbors using broadcast. Even though the requester vehicles find the RC in the current cell, there is a high probability that the number of cluster members in the cell is not enough to provide the requested resource to the requester vehicles when the vehicles are sparse. Therefore, the requester vehicle needs to wait until it enters the next cell, and it causes a lot of delays. In the proposed scheme, since the requester vehicle can use all the vehicles in the entire network area, it has enough cluster members. Furthermore, the requester vehicles do not have to broadcast to find the RC because vehicles already know neighbors’ information about the resource. For these reasons, the requester vehicles suffer very few delays in finding the RC. When vehicles are dense, the RC has many member vehicles to be allocated to the requester vehicles. Furthermore, in the entire network, since there is much congestion when vehicles are dense because vehicles exchange their information with their neighbors, the requester vehicles suffer an additional slight delay.

Figure 5b shows the resource searching delay according to the ratio of the requester vehicles. If there are many requested vehicles, they should distribute limited resources appropriately. In SERVitES, only vehicles that are in the same cell are considered cluster members. Thus, if the cell does not have enough resources to provide the service to the requester vehicle, the requester vehicle has to move to another cell. When the number of the requester vehicles is increased, the requester vehicle has a low probability of getting enough resources because the amount of the resources that has to be shared is fixed within the same cell. That is the reason that the resource searching delay increases when there are many requester vehicles in SERVitES. In the proposed scheme, even though the number of requester vehicles increases, it has enough resources because the RCs use the entire network. Therefore, the ratio of the requester vehicles does not affect the resource searching delay in the proposed scheme.

Figure 5c shows the resource searching delay according to the average speed of vehicles. The speed of vehicles affects the connectivity between vehicles. However, in SERVitES, the requester vehicles are more affected by the delay until entering the next cell. When the speed of the requester vehicle is fast, that delay can be reduced. Furthermore, the speed of vehicles affects new chances to meet other vehicles in the cell. If vehicles are fast, the requester vehicle can contact many vehicles in the cell. This makes the requester vehicle find the RC faster, so the delay is reduced. In the proposed scheme, the number of vehicles that can cluster member vehicles does not change because the RC uses all vehicles in the entire network area, regardless of the speed of the vehicle. Therefore, the average speed of vehicles does not affect the resource searching delay in the proposed scheme.

Figure 5d shows the resource searching delay according to the number of the considered resource types. The larger number of the considered resource types has more distributed RCs. In SERVitES, the cluster is limited to a maximum of 3 hops, requiring more distributions of RCs. Since the small-size RC due to the hop limitation can hold a small amount of resources, the requester vehicles might not be served enough resources. In that case, since the requester vehicle should find another RC to satisfy its requested resource, SERVitES has more delays than the proposed scheme when the number of the considered resource types is 1. In the proposed scheme, if more RCs are distributed, the requester vehicle might not find the RS in 1 hop. In this case, the RC allocates the multi-hop member provider vehicle to the requester vehicle. When the number of the considered resource types increases, RCs are more distributed, and it denotes that the requester vehicle needs more hops to receive the service. Therefore, it causes an additional slight delay according to hops between the requester vehicle and the provider member vehicle.

#### 5.2.2. The Number of Packets

The proposed scheme does not use broadcast or flood to find the RC. Compared with the proposed scheme, in SERVitES, since the requester vehicle and its neighbors do not know about other neighbors’ information, the requester vehicle finds the cluster member of the requested resource by flooding the request message. Furthermore, every time the requester vehicle needs a new cluster due to leaving the vehicular cluster or entering the next cell, it has to find the cluster member again. Because it generates a lot of packets to search for the new vehicular cluster, the proposed scheme spends fewer packets than SERVitES.

**Figure 6:**
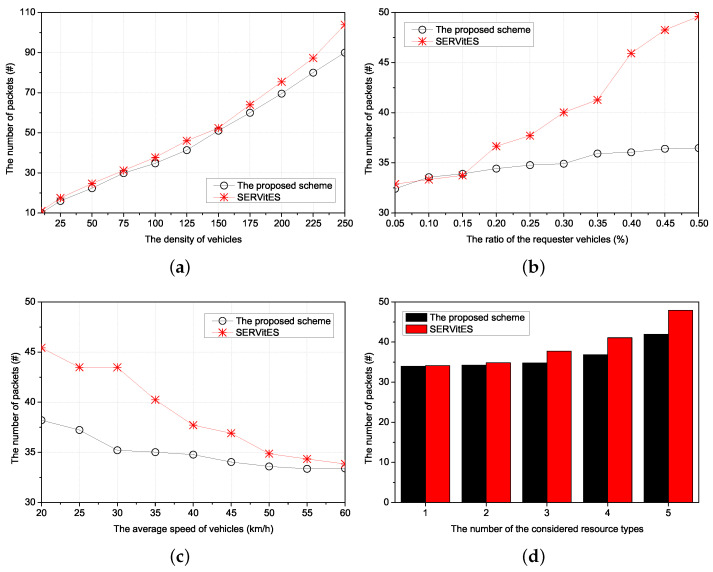
The number of packets according to (**a**) the density of vehicles, (**b**) the ratio of the requester vehicles, (**c**) the average speed of vehicles, and (**d**) the number of the considered resource types.

Figure 6a shows the number of packets according to the density of vehicles. The requester vehicle sends a request message when it needs additional resources. After receiving the request message, neighboring vehicles of the requester vehicle reply with information about the resource and mobility to the requester vehicle. If there are many neighbors of the requester vehicle, the requester vehicle receives more reply messages to obtain information about neighbors. It causes an increased number of packets. In SERVitES, the requester vehicle can know about up to 3 hop neighbors via flooding. In addition, if the requester vehicle can not find enough resources in the cell, it repeats requesting for the resource and receiving reply messages in the next cell. Since flooding and the repeated request process cause a lot of packets, SERVitES spends more packets than the proposed scheme to search the RC. When vehicles are dense, the proposed scheme also has a lot of packets because the requester vehicle receives more reply messages. However, the requester vehicle in the proposed scheme does not use flooding because all vehicles know the resource and mobility information of their neighbors. The join message by the requester vehicle is forwarded to the CH of the same or different resource types. Therefore, the proposed scheme spends smaller packets than SERVitES.

Figure 6b shows the number of packets according to the ratio of the requester vehicles. If there are many requester vehicles, the total size of the requested resources is dramatically increased. Since the total available resources of the RC are limited, the later requester vehicles might not receive enough resources. In SERVitES, the later requester vehicles that cannot receive enough resources from the RC have to move to the next cell. If more vehicles request resources, the number of packets that are generated in the next cell is increased because they do have not enough resources. On the other hand, if the requester vehicles are few, the requester vehicle can satisfy the service in this cell because the cell has enough resources to provide. Furthermore, SERVitES has a few packets when the requester vehicles are few because it just spends 1 hop broadcast to find the RC. In the proposed scheme, when the requester vehicles are increased, the later requester vehicle cannot use a good cluster member vehicle as a provider. The later requester vehicles have to spend more hops to receive the service, and the increased number of hops causes more packets. Therefore, the number of packets is increased by the increased number of requester vehicles in the proposed scheme. However, because the RC has enough resources as there are no limitations of hops and the requester vehicles do not need to flood to find the RC, the proposed scheme has a smaller number of packets than SERVitES.

Figure 6c shows the number of packets according to the average speed of vehicles. The fast vehicle spends more packets to maintain the service because it has weak connectivity, but because we only consider the packets to search the RC, the number of packets is decreased due to the allocation of vehicles with small hops when the vehicle’s speed increases. In SERVitES, when the average speed of vehicles is slow, the requester vehicle can use the vehicles that have more hops from the requester vehicle because the connectivity between vehicles is stable. Since the requester vehicle that has the small hops provider spends a small number of packets, it spends a smaller number of packets when the vehicles are fast. Because the requester vehicles can use only 1 hop neighbors when the vehicles are very fast, the performance of the two schemes becomes similar. However, because of the characteristics of the proposed scheme, the number of packets to search the RC in the proposed scheme is less than SERVitES.

Figure 6d shows the number of packets according to the number of the considered resource types. If the number of the considered resource types is 1, all RCs have the same type of resource. If many types of resources are considered, RCs have to be distributed according to their types. Then, the RCs have a reduced number of member vehicles because of the distribution. The reduced number of member vehicles in an RC causes additional hops to find enough resources for the requester vehicles. In SERVitES, if the provision of resources of the RC is not enough in the cell, the requester vehicle has to repeat the request process in the next cell. Because of the additional hops and a lack of the provided resources in an RC, the requester vehicles spend more packets to find the RC. For this reason, more of the considered resource types make the number of packets to find the RC increase. In the proposed scheme, the lack of provided resources is solved because the requester vehicle can use all the vehicles in the entire network area and the number of hops is not limited. In addition, because all the vehicles do not need to use broadcast to find the RC, the requester vehicles can reduce the number of packets. As a result, it derives a smaller number of packets than SERVitES.

#### 5.2.3. The Success Ratio

The proposed scheme does not have a separate area to operate the cluster. Compared with the proposed scheme, in SERVitES, only vehicles within the same cell as the requester vehicle can be considered members of the RC. Since the restricted searching area has limited resources to find the RC, there might not be enough resources to meet the requested resources. Therefore, the proposed scheme improves the success ratio more than SERVitES.

**Figure 7:**
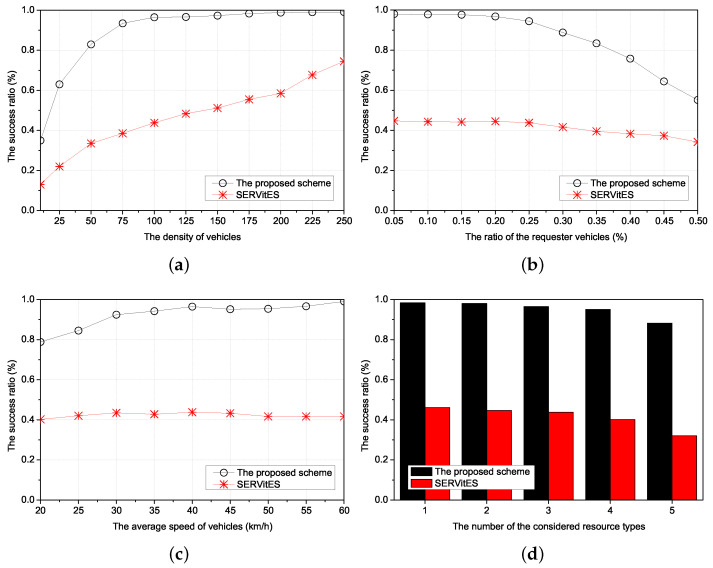
The success ratio according to (**a**) the density of vehicles, (**b**) the ratio of the requester vehicles, (**c**) the average speed of vehicles, and (**d**) the number of the considered resource types.

Figure 7a shows the success ratio according to the density of vehicles. When the density of vehicles is high, the number of vehicles that can be members of the vehicular cluster is increased. Since the number of members in the vehicular cluster affects the amount of provision resources, the dense number of vehicles improves the success ratio for searching the RC. In SERVitES, the number of vehicles that can be used is restricted because of the limited searching coverage of the same cell with the requester vehicle. Therefore, when the vehicles are not dense, the cell does not have enough resources and vehicles to provide the requested resources to the requester vehicle. In addition, the requester vehicles can use the RC within three-hop neighbors. In the proposed scheme, if the vehicles are very sparse, the network might have insufficient resources to provide the service. Nevertheless, because there is no limitation on the range to search the RC, the proposed scheme has a higher success ratio than SERVitES.

Figure 7b shows the success ratio according to the ratio of the requester vehicles. The ratio of the requester vehicles denotes the number of the requester vehicles in the network area. If there are so many requester vehicles, the resources in the network will not be enough to meet all the requester vehicles’ needs. So, the increased number of requester vehicles makes the success ratio drop. In SERVitES, the vehicles that can be used as a provider are needed more when the number of the requester vehicles is increased, but because the number of vehicles in the same cell with the requester is limited, there is a lack of providing resources and failure to search the RC. In the proposed scheme, if half of the vehicles want the resources, the failure to search the RC is increased because the resources in the entire network are not enough to meet that request. However, the resources that can be used are enough to make provision for the requester vehicles because there is no limitation on the search coverage other than the comparing scheme.

Figure 7c shows the success ratio according to the average speed of vehicles. If the vehicle is fast, the vehicle can be connected very quickly to another vehicle. Furthermore, new vehicles continue to flow into the search area. In these cases, the success ratio rises because the number of vehicles that can be member vehicles is raised. In SERVitES, the new connection does not have much impact on RC searches due to hop restrictions on the requester vehicles. However, in the proposed scheme, when the vehicles are very fast, the new connection is increased, and it affects the success ratio to search the RC. In terms of the search success ratio, the performance is improved because the high speed of vehicles is a good chance to contact other vehicles.

Figure 7d shows the success ratio according to the number of the considered resource types. The number of the considered resource types denotes the distribution of the RCs. The many distributions of RCs in the fixed number of vehicles lead to a reduced amount of the provided resources, which affects the success ratio of finding the RC. Because of the searching area limitations in SERVitES, it has a smaller number of vehicles that can be member vehicles of the RC. In addition, the distributions of RCs make the problem worse. The proposed scheme has the same problem as SERVitES in terms of the distributions of the RCs. However, because of its extended searching area compared to SERVitES, the requester vehicles in the proposed scheme have enough resources.

## 6. Conclusions

Vehicular clouds are considered an attractive approach because vehicles collaborate using their resources to create value-added services such as safety and entertainment applications. For constructing vehicular clouds, an efficient resource search and allocation process is one of the most important and challenging issues for supporting various vehicular cloud services. To search and allocate sufficient available resources for the V2V cloud construction, clustering techniques based on the hierarchical structure are utilized in VANETs. However, since they construct clusters of vehicles for managing the resource information by using the proximity between vehicles such as the same road or region units, they cannot rapidly search and stably allocate resources for vehicles with various resource types and different mobility states. As a result, they cannot satisfy various vehicular cloud services. Therefore, we propose a Resource Cluster-based Resource Search and Allocation (RCSA) scheme based on resource clusters to efficiently search and allocate resources. RCSA constructs vehicles with the same resource type as a resource cluster and selects the cluster member vehicle with the longest average connection time and the most resources as a Cluster Header (CH). In resource clusters of various resource types, RCSA supports both an intra-resource search for the same resource type and an inter-resource cluster search for the different resource types, respectively. Results of simulation conducted in various environments verify that RCSA achieves better performance than the existing schemes in terms of the resource searching delay, the number of packets, and the success ratio. We propose to construct and manage a resource cluster by merging the resources of vehicles based on their resource type using V2V communication to reduce the burden on infrastructure. By utilizing vehicles that have various resources such as storage, bandwidth, RAM, CPU, etc., resources can be allocated according to the resource needs of the requester vehicle to meet its requirements. However, a vehicle’s resource needs may not require a single type of resource but may require multiple types of resources. Therefore, it is necessary to be able to construct and manage resource clusters with multiple resource types. In future work, we will study how to manage and optimize multi-type resource clusters with different types of resource needs and available resources.

## Figures and Tables

**Table 1:** Simulation Parameter Settings.

Parameter	Values
Network size	2 km × 2 km
Vehicle density	[50, 250]/km^2^
Vehicle average speed	[20, 60] km/h
V2I communication range	up to 1000 m
V2V communication range	up to 200 m
V2I transmission rate	up to 54 Mbps
V2V transmission rate	up to 54 Mbps
Vehicle cache storage size	512 GB
RSU cache storage size	1 TB
The distance between RSUs	1 km
Vehicle mobility model	Manhattan model
The number of resource types	[1, 5]
The requester vehicle ratio	[5, 50]%

## Data Availability

Data are contained within the article.
